# Inhibitory Effects of Eucalyptus and Banaba Leaf Extracts on Nonalcoholic Steatohepatitis Induced by a High-Fructose/High-Glucose Diet in Rats

**DOI:** 10.1155/2015/296207

**Published:** 2015-04-27

**Authors:** Yoshihisa Takahashi, Keiichiro Sugimoto, Yurie Soejima, Arisa Kumagai, Tatsuki Koeda, Aiko Shojo, Kazuya Nakagawa, Naoki Harada, Ryoichi Yamaji, Hiroshi Inui, Toshikazu Yamanouchi, Toshio Fukusato

**Affiliations:** ^1^Department of Pathology, Teikyo University School of Medicine, Tokyo 173-8605, Japan; ^2^Research and Development Center, Nagaoka Perfumery Co. Ltd., Ibaraki, Osaka 567-0005, Japan; ^3^Center for Research and Development of Bioresources, Osaka Prefecture University, Sakai, Osaka 599-8531, Japan; ^4^Division of Applied Life Sciences, Graduate School of Life and Environment Sciences, Osaka Prefecture University, Sakai, Osaka 599-8531, Japan; ^5^Department of Nutrition and Health, Faculty of Human Development, Soai University, Suminoe-ku, Osaka 559-0033, Japan; ^6^Department of Clinical Nutrition, College of Health and Human Sciences, Osaka Prefecture University, Habikino, Osaka 583-8555, Japan; ^7^Department of Internal Medicine, Teikyo University School of Medicine, Tokyo 173-8605, Japan

## Abstract

Nonalcoholic steatohepatitis (NASH) is a liver disease associated with metabolic syndrome. The aim of this work was to examine whether eucalyptus (*Eucalyptus globulus*) leaf extract (ELE) and banaba (*Lagerstroemia speciosa* L.) leaf extract (BLE) inhibited NASH induced by excessive ingestion of fructose in rats. Wistar rats were divided into four groups according to four distinct diets: starch diet (ST), high-fructose/high-glucose diet (FG), FG diet supplemented with ELE, or FG diet supplemented with BLE. All rats were killed after 5 weeks of treatment. Serum alanine aminotransferase and total cholesterol levels were significantly lower in the BLE group than in the FG group. Liver histopathology, including steatosis, lipogranulomas, and perisinusoidal fibrosis, was significantly attenuated in the ELE and BLE groups compared with the FG group. Levels of 2-thiobarbituric acid reactive substances (TBARS), which reflect oxidative injury to the liver, were significantly suppressed by ELE and BLE. Western blotting analysis indicated that interleukin-6 expression levels were significantly lower in the ELE and BLE groups than in the FG group. These results suggest that ELE and BLE reduced lipogenesis, oxidative stress, and inflammatory cytokine expression and thus inhibited NASH induced by excessive ingestion of fructose in rats.

## 1. Introduction

Nonalcoholic fatty liver disease (NAFLD) is a condition in which excessive fat (primarily triacylglycerols (TAG)) accumulates in the liver of a patient without a history of alcohol abuse [1]. The histological spectrum of NAFLD pathology includes simple steatosis and nonalcoholic steatohepatitis (NASH), which is characterized by lobular inflammation and hepatocellular injury, as well as hepatic steatosis. NASH is a progressive disease that can advance to liver cirrhosis and hepatocellular carcinoma [2, 3]. NAFLD/NASH is recognized as a hepatic manifestation of metabolic syndrome [4, 5]. Notably, the disorder is a growing clinical and public health concern, as the prevalence of NAFLD/NASH is rapidly increasing worldwide due to the increased rate of obesity. As a result, it is currently the most common chronic liver disease [6, 7].

Excessive consumption of fructose, largely resulting from the rapid increase in the amount of high-fructose corn syrups (HFCSs) in the human diet, is considered to be one of the major factors contributing to the increasing rate of obesity and metabolic syndrome [8, 9]. Our group and others have shown that fructose-enriched diet causes metabolic syndrome and NAFLD/NASH in experimental animals [10–12]; therefore, fructose enrichment has become a common nutritional animal model of NAFLD/NASH. It has been reported that the amount of fructose consumption is higher in patients with NAFLD and that their hepatic ketohexokinase activity, which plays a crucial role in fructose metabolism in the liver, is elevated compared to healthy subjects [13].

Eucalyptus (*Eucalyptus globulus*) is an evergreen tree native to Australia, which is widely distributed around the world. The leaves of this plant are used as a traditional remedy for diabetes mellitus in South America and Africa, and its antihyperglycemic effect has been demonstrated in streptozotocin-induced diabetic mice [14]. We have previously shown that eucalyptus leaf extract (ELE) inhibits intestinal fructose absorption and suppresses the accumulation of hepatic TAG induced by the excessive ingestion of fructose in rats [15]. This inhibitory effect on intestinal fructose absorption has also been observed in human subjects [16]. Banaba (*Lagerstroemia speciosa* L.) is another folk medicine used to treat diabetes mellitus in various parts of the world, primarily Southeast Asia, and many clinical and experimental studies have confirmed its antihyperglycemic effect [17].

In the present study, we examined potential inhibitory effects of ELE and banaba leaf extract (BLE) on NASH induced by excessive ingestion of fructose in rats. We report that ELE and BLE inhibited the development and progression of hepatic lesions in our animal model of NAFLD/NASH. These effects were associated primarily with decreased lipogenesis, presumably due to the suppression of intestinal fructose absorption. In addition, assays for inflammation and oxidative stress suggested that the antioxidative and anti-inflammatory effects of ELE and BLE are important mediators of NASH inhibition.

## 2. Materials and Methods

### 2.1. Ethics Statement

This study was carried out in strict accordance with the recommendations of the Guide for the Care and Use of Laboratory Animals of the National Institutes of Health. The protocol was approved by the Animal Care and Use Committee of Osaka Prefecture University (permit number: 21-2). All the animals received humane care, and all efforts were taken to minimize suffering.

### 2.2. Preparation of ELE and BLE

Dried eucalyptus and banaba leaves were purchased from K. Kobayashi & Co., Ltd. (Kobe, Japan) and were extracted with boiling ethanol-water (1 : 2, v/v) under reflux for 2 h. The extract was then filtered and evaporated to dryness* in vacuo*.

### 2.3. Animals and Experimental Protocols

Thirty 5-week-old male Wistar rats were purchased from Kiwa Laboratory Animals Co., Ltd. (Wakayama, Japan), and maintained on a starch diet for 1 week. Then, rats were divided into four groups according to diet: starch (ST) (*n* = 7), high-fructose/high-glucose (FG) (*n* = 9), FG diet supplemented with ELE (*n* = 7), and FG diet supplemented with BLE (*n* = 7). The latter two groups were termed ELE and BLE, respectively. The rats in the ST and FG group were fed a starch and FG diet* ad libitum*, respectively. The ELE and BLE groups were fed identically to the FG group, but their feed was supplemented with 1% (w/w) ELE or BLE. The composition of each diet is shown in Table 1. All rats were housed individually in a room with controlled temperature (23 ± 2°C), humidity (60 ± 10%), and light cycles (09:00–21:00). The diets were stored in a refrigerator at 4°C. The feed containers were refilled with fresh diet 3 times a week, and food consumption was recorded.

The rats were killed at 11 weeks of age, 5 weeks after commencing the diet. The rats were starved for 16 h, and their body weights were measured before killing them. Rats were starved overnight to avoid influences of food consumption on serum glucose and insulin levels. After the rats were anesthetized with isoflurane, blood samples from each rat were collected by cardiac puncture, and the serum was separated by centrifugation. The liver of each rat was excised and weighed, and samples were collected for histological analysis and snap freezing. The weight of epididymal adipose tissue (EAT) was also measured.

### 2.4. Biochemical Analysis of Serum

Serum aspartate aminotransferase (AST), alanine aminotransferase (ALT), alkaline phosphatase (ALP), cholinesterase (ChE), total cholesterol (T-Cho), high density lipoprotein (HDL-Cho), and glucose levels were determined by routine methods using the Hitachi 7700 Series (DDP) autoanalyzer (Hitachi High-Technologies Corporation, Tokyo, Japan). Arteriosclerotic index (AI) was calculated as (T-Cho − HDL-Cho)/HDL-Cho. Serum insulin and adiponectin levels were measured by enzyme-linked immunosorbent assay (ELISA) using the rat insulin ELISA kit (RTU) (Shibayagi Co., Ltd., Shibukawa, Japan) and mouse/rat adiponectin ELISA kit (Otsuka Pharmaceutical Co., Ltd., Tokyo, Japan), respectively.

### 2.5. Histological Analysis

The central part of the largest liver lobe was fixed in 10% formaldehyde solution and processed for light microscopy by standard methods. The largest whole section was histologically evaluated. Azan-Mallory staining was performed in addition to hematoxylin and eosin staining to assess hepatic fibrosis. Histopathological features of steatohepatitis were evaluated semiquantitatively according to the validated histological scoring system of Kleiner et al. [18]. The degree of macro- and microvesicular steatosis was evaluated by the percentage of hepatocytes containing macro- and microvesicular fat, respectively, and graded as follows: grade 0 (<5%), grade 1 (5–33%), grade 2 (>33–66%), and grade 3 (>66%). Lobular inflammation was classified as follows: 0 (no foci), 1 (<2 foci per 200x field), 2 (2–4 foci per 200x field), or 3 (>4 foci per 200x field). Lipogranulomas were evaluated as follows: 0 (no foci), 1 (<1 foci per 200x field), 2 (1-2 foci per 200x field), or 3 (>2 foci per 200x field). Portal inflammation was graded as follows: 0 (none: no lymphocytes observed), 1 (mild: sparse lymphocytes present in some or all portal tracts), 2 (moderate: denser lymphocytic infiltration in most portal tracts), or 3 (severe: dense lymphocytic infiltration in most or all portal tracts). Portal fibrosis was evaluated as follows: 0 (none), 1 (mild: portal expansion), 2 (moderate: portal fibrosis with septa), 3 (severe: portal-portal or portal-central bridging septa without regenerative nodules), or 4 (cirrhosis). Intralobular perisinusoidal fibrosis was observed mainly in the periportal area and noted as follows: 0 (none), 1 (mild), or 2 (moderate). Fibrosis staging was classified as follows: 0 (none), 1 (perisinusoidal or portal), 2 (perisinusoidal and portal), 3 (bridging fibrosis), or 4 (cirrhosis).

### 2.6. Determination of TAG Content in the Liver

We measured TAG content in the liver to confirm the extent of hepatic steatosis in each experimental group. TAG in the liver was extracted according to the method described by Folch et al. [19] and quantified using a commercially available kit (L-type TG·H) (Wako Pure Chemical Industries, Ltd., Osaka, Japan) according to the manufacturer's instructions.

### 2.7. 2-Thiobarbituric Acid Reactive Substances (TBARS) Levels in the Liver

To assess oxidative injury in the liver, we measured the hepatic TBARS levels. The liver was homogenized with a polytron homogenizer in 1.15% KCl at 4°C. TBARS levels in the homogenate were determined using the OXItec TBARS assay kit (ZeptoMetrix, NY, USA) according to manufacturer instructions.

### 2.8. Determination of Glucose-6-Phosphate Dehydrogenase (G6PDH)

The liver was homogenized with a polytron homogenizer in 25 mmol/L HEPES-KOH buffer of pH 7.4, containing 0.15 mol/L KCl at 4°C. After centrifugation at 10,000 g for 20 min, we obtained supernatant for use as a crude solution containing active liver enzymes. G6PDH activity was measured as described previously [20]. Protein concentration was determined by the Bradford method [21], and activity of G6PDH was normalized to the protein concentration.

### 2.9. Western Blotting

We examined protein expression levels of inflammatory cytokines and cytokine receptor genes in the liver by Western blotting to elucidate molecular mechanisms of the effects of ELE and BLE. Liver samples were homogenized and centrifuged at 10,000 g at 4°C for 15 min, and the protein concentration in each was determined using a NanoDrop 2000 spectrophotometer (Thermo Fisher Scientific, Waltham, MA, USA). Aliquots of 50 *μ*g protein were resolved by electrophoresis on 12.5% SDS-PAGE gels and transferred onto polyvinylidene fluoride membranes. These membranes were incubated in blocking buffer (5% nonfat milk powder in phosphate buffered saline (PBS)) for 1 h followed by incubation with primary antibodies in 5% bovine serum albumin (BSA) in PBS overnight at 4°C, with gentle agitation. The primary antibodies used for Western blotting were as follows: Tumor Necrosis Factor- (TNF-) *α* (1 : 250 dilution, R&D Systems, Minneapolis, MN, USA), TNF Receptor 1 (TNFR1) (1 : 50 dilution, MBL, Nagoya, Japan), interleukin- (IL-) 6 (1 : 200 dilution, Santa Cruz Biotechnology, Dallas, TX, USA), and Monocyte Chemotactic Protein- (MCP-) 1 (1 : 500 dilution, Abcam, Cambridge, UK). As a loading control, blots were incubated with antibodies against *β*-actin (1 : 1000 dilution, Santa Cruz Biotechnology). The membranes were subsequently washed with 0.1% Tween-20 in PBS and incubated with anti-rabbit or anti-goat secondary antibodies (each 1 : 2000 in 0.1% Tween-20 in PBS) for 1 h at room temperature. The blots were again washed with 0.1% Tween-20 in PBS, and the expression of antibody-linked protein was determined using ECL Western Blotting Detection Reagents (Amersham Pharmacia Biotech Inc., NJ, USA). The optical density of the bands was quantified by ImageQuant software (GE Healthcare Life Sciences, Little Chalfont, UK) and reported in arbitrary units. The protein expression level of each gene was normalized by the expression level of *β*-actin.

### 2.10. Statistics

For continuous variables, data are presented as mean ± standard deviation, and a one-way analysis of variance (ANOVA) followed by Dunnett's post hoc test was performed to assess the significance of the differences. For semiquantitative data obtained by histological assessment, data are presented as the median (min. to max.), and a Kruskal-Wallis test followed by Steel's post hoc test was performed to determine statistical significance. *P* < 0.05 was considered statistically significant.

## 3. Results

### 3.1. General Observations

No rats died during the experiment.  Table 2 shows data detailing food consumption levels, calorie intake, and body, liver, and EAT weights of rats in each group. Food consumption levels were lower in the FG, ELE, and BLE groups than in the ST group, and this difference was statistically significant for the ELE and BLE groups. Calorie intake also tended to be lower in the FG, ELE, and BLE groups than in the ST group. Body weight was found to be significantly lower in the FG, ELE, and BLE groups than in the ST group. Liver weight was higher in the FG group than in the ST group. In contrast, liver weight was lower in the ELE and BLE groups than in the FG group, and this difference was statistically significant for the BLE group. Liver/body weight ratio was significantly higher in the FG group than in the ST group, while it was significantly lower in the BLE group than in the FG group. EAT weight was higher in the FG group than in the ST group, and it was significantly lower in the ELE and BLE groups than in the FG group. EAT/body weight ratio was also higher in the FG group than in the ST group. EAT/body weight ratio was lower in the ELE and BLE groups than in the FG group, and the difference was statistically significant for the ELE group.

### 3.2. Biochemical Data for Serum

Table 3 shows the data obtained through biochemical analysis of serum obtained from each group. ALT levels were significantly higher in the FG group than in the ST group, while they were lower in the ELE and BLE groups than in the FG group. This difference was statistically significant for the BLE group. T-Cho levels were higher in the FG group than in the ST group. They were lower in the ELE and BLE groups than in the FG group, and the difference was statistically significant for the BLE group. Similarly, AI was higher in the FG group than in the ST group; it was lower in the ELE and BLE groups than in the FG group and the difference was statistically significant for the BLE group. Assays for AST, ALP, ChE, HDL-Cho, glucose, insulin, and adiponectin levels revealed no significant differences among the experimental groups.

### 3.3. Histological Findings

**Figure 1:**
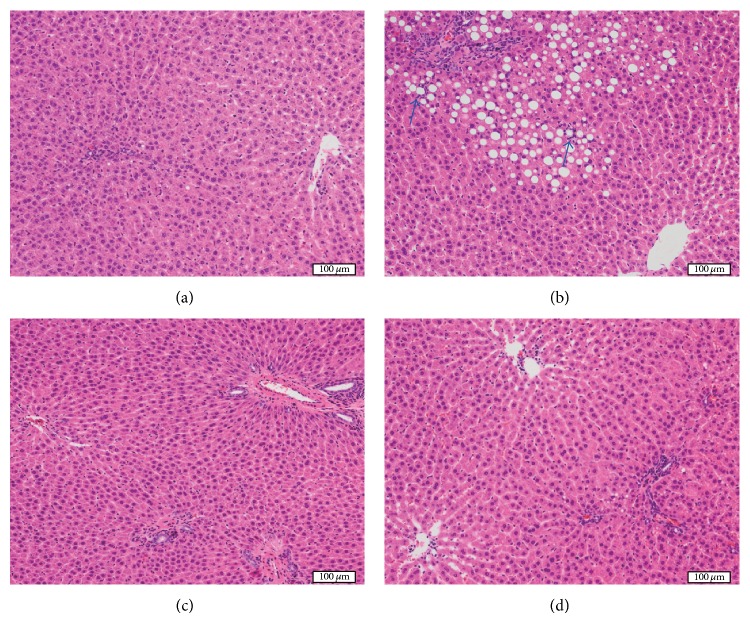
Histological appearance of the liver. Although rats in the ST group show only mild steatosis and inflammation (a), rats in the FG group show marked steatosis and scattered foci of lobular inflammation (arrows) (b). Steatosis is markedly alleviated in rats of the BLE (c) and ELE (d) groups.

Differences in the histological appearance among liver lobules were not conspicuous in any of the rats. Although rats in the ST group showed only mild steatosis and inflammation, rats in the FG group showed liver histopathology consistent with NASH (Figures 1(a) and 1(b)). Steatosis and perisinusoidal fibrosis in the FG group was mainly distributed in zone 1.  Table 4 summarizes the histological findings of each group. The grade of macrovesicular steatosis was significantly higher in the FG group than in the ST group. In keeping with ELE and BLE preventing NASH-related pathologies, macro- and microvesicular steatosis were significantly lower in the ELE and BLE groups than in the FG group (Figures 1(c) and 1(d)). The grade of microvesicular steatosis in the BLE group was also found to be significantly lower than that of the ST group.

Lobular inflammation tended to be milder in the BLE group than in the FG group, but statistically significant differences were not observed among any experimental groups. With regard to portal inflammation, there were no significant differences among the experimental groups. The number of lipogranulomas was significantly higher in the FG group than in the ST group, and the granuloma number was lowered by ELE and BLE administration compared to the FG group.

We further tested the effects of ELE or BLE treatment on the development of various forms of fibrosis in response to an FG diet. The degree of portal fibrosis was significantly more severe in the FG group than in the ST group and it was lower in the ELE and BLE groups than in the FG group, but the differences were not statistically significant. While the degree of perisinusoidal fibrosis was significantly more severe in the FG group than in the ST group, it was significantly milder in the ELE and BLE groups than in the FG group. Fibrosis stage was significantly higher in the FG and ELE groups than in the ST group. Fibrosis stage also tended to be lower in the ELE and BLE groups than in the FG group, but the differences were not statistically significant.

### 3.4. TAG Content, TBARS Levels, and G6PDH Activity in the Liver

**Figure 2:**
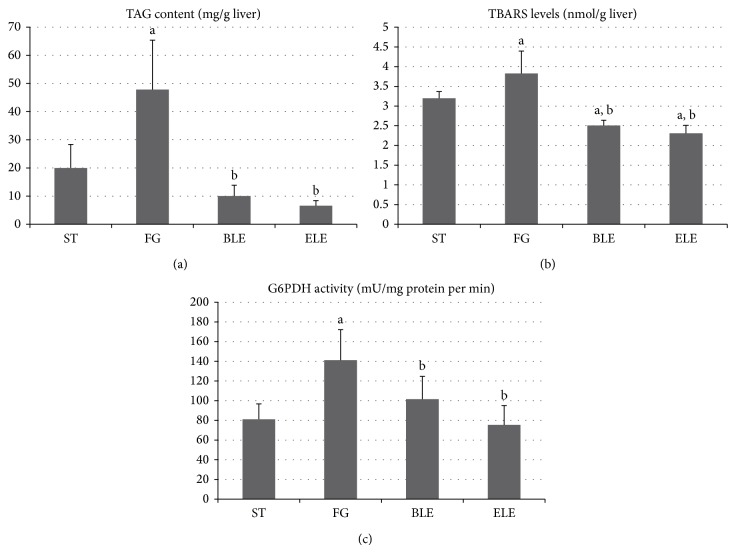
TAG content, TBARS levels, and G6PDH activity in the liver. TAG content (a), TBARS levels (b), and G6PDH activity (c) in the liver are significantly higher in the FG group than in the ST group, and they are significantly lower in the ELE and BLE groups than in the FG group. TBARS levels in the ELE and BLE groups are also significantly lower than those in the ST group. ^a^Significantly different from the ST group (*P* < 0.05). ^b^Significantly different from the FG group (*P* < 0.05).

TAG content, TBARS levels, and G6PDH activity in the liver were significantly higher in the FG group than in the ST group, and they were significantly lower in the ELE and BLE groups than in the FG group (Figure 2). TBARS levels in the ELE and BLE groups were also found to be significantly lower than those in the ST group.

### 3.5. Protein Expression Levels of Inflammatory Cytokine and Receptor Genes

**Figure 3:**
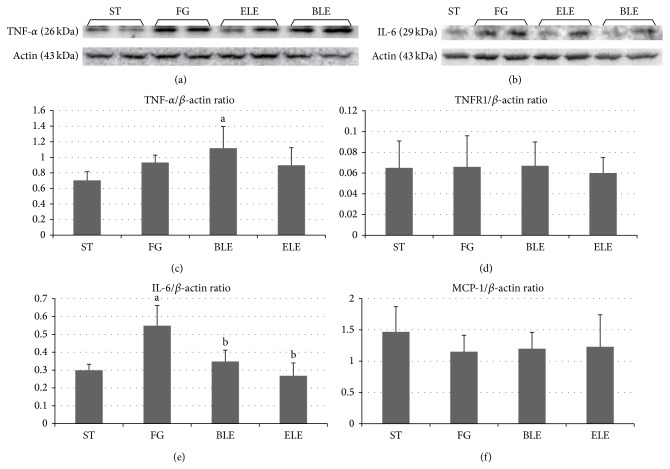
Protein expression levels of inflammatory cytokine and receptor genes determined by Western blotting. IL-6 expression levels are significantly higher in the FG group than in the ST group, and they are significantly lower in the ELE and BLE groups than in the FG group ((b), (e)). TNF-*α* ((a), (c)), TNFR1 (d), and MCP-1 (f) expression levels did not show significant differences among the experimental groups, with the exception of significantly higher TNF-*α* expression in the BLE group than in the ST group. ^a^Significantly different from the ST group (*P* < 0.05). ^b^Significantly different from the FG group (*P* < 0.05).

Figure 3 shows Western blotting results revealing the protein expression levels of the inflammatory cytokines or receptors: TNF-*α*, TNFR1, IL-6, and MCP-1. IL-6 expression levels were significantly higher in the FG group than in the ST group, and they were significantly lower in the ELE and BLE groups than in the FG group. With regard to TNF-*α*, TNFR1, and MCP-1 expression levels, there were no significant differences among the experimental groups, with the exception of significantly higher TNF-*α* expression in the BLE group than in the ST group.

## 4. Discussion

In the present study, we have shown that ELE and BLE attenuate NASH induced by a fructose-enriched diet in rats. To the best of our knowledge, this is the first study to examine the preventative effects and potential therapeutic benefits of ELE and BLE on NAFLD/NASH in detail.

There are a variety of established nutritional animal models of NAFLD/NASH [22]. We focused on NAFLD/NASH induced by excessive intake of fructose. Although the high-fructose diet model of NASH is well established [10–12, 22], in this study, we used a high-fructose/high-glucose diet to render the experimental diet more similar to the human diet. HFCSs, which are used as sweeteners and thought to substantially contribute to the increasing prevalence of obesity and metabolic syndrome [23, 24], contain glucose as well as fructose. Indeed, the levels of fructose and glucose contents in most foodstuffs are practically identical. As evidence that our method is an appropriate animal model of NASH, liver histopathology similar to NASH and increased serum ALT level were induced in rats as a result of a high-fructose/high-glucose diet. Steatosis and perisinusoidal fibrosis in our model was predominant in zone 1, in contrast to typical forms of NAFLD/NASH in adult humans. We obtained similar findings in a previous study using a pure high-fructose diet [12]. Thus, this pattern may be the characteristic of NAFLD/NASH caused by excessive consumption of fructose. Unexpectedly, body weight in the FG group was significantly lower than that of the ST group. This phenomenon is most probably explained by our observation that food consumption and calorie intake in the FG group tended to be lower than those in the ST group. This was probably due to the taste of the FG diet, which was less preferred compared to the ST diet. We designed the ST diet to be tasty; however, the FG diet contained large amount of fructose and glucose, which might have made it too sweet. Furthermore, in our previous study, the energy intake in the high-fructose diet group was found to be significantly lower than that in the starch diet group [12]. Food consumption tended to be lower in the ELE and BLE groups than in the FG group, although the differences were not statistically significant. Possibly, the addition of ELE and BLE resulted in a further taste loss of the diet. However, EAT weight in the FG group was higher than that in the ST group; this result was consistent with abdominal obesity typically associated with metabolic syndrome.

Liver weight and liver/body weight ratios were significantly lower in the BLE group than in the FG group, consistent with amelioration of NASH. We determined that the decrease in liver weight was not due to hepatotoxicity, as liver injury was not observed upon histological assessment. Serum levels of ALT, T-Cho, and AI were significantly lower in the BLE group than in the FG group. This suggests that BLE administration significantly improved hypercholesterolemia and hepatocellular injury. Although the differences were not statistically significant, these serological data tended to be lower in the ELE group than in the FG group as well, suggesting there may be a similar, if perhaps not as robust, effect of ELE. Statistically significant differences in the serum levels of AST and ALP were not observed among the experimental groups. However, these serological markers are less specific to liver cell injury than ALT.

Evaluation of liver histopathology revealed that macro- and microvesicular steatosis, lipogranulomas, and perisinusoidal fibrosis were significantly milder in both ELE and BLE groups than in the FG group. These data indicate that ELE and BLE attenuated pathological findings of NASH. Because the largest whole section of the largest liver lobe was examined and heterogeneity among liver lobules was not remarkable, the observed histological findings likely reflected the whole aspect of the liver. A significant inhibitory effect of ELE and BLE on liver steatosis was further confirmed by quantification of TAG content in the liver.

Fructose absorbed in the small intestine is transported mainly to the liver and metabolized to triose phosphates by the action of ketohexokinase, aldolase B, and triokinase without the participation of 6-phosphofructokinase, which is the main rate-controlling step in glycolysis [25]. Therefore, fructose can serve as a relatively unregulated acetyl-CoA and is more lipogenic than glucose. Lipogenic enzymes such as G6PDH and fatty acid synthase are activated and hepatic TAG concentration is significantly elevated after excessive consumption of sucrose or fructose [26, 27].

We previously reported that ELE inhibits intestinal fructose absorption [15]. A decrease in fructose absorption would result in the reduction of lipogenesis in the liver. Indeed, hepatic G6PDH activity was significantly lower in the ELE group than in the FG group, and only very small amounts of TAG accumulated in the liver of the ELE group, in contrast to the FG group. When considering these and our previous study, we speculated that ELE prevents NASH induced by the excessive ingestion of fructose mainly by decreasing its intestinal absorption. BLE also reduced lipogenesis and prevented NASH in rats fed with the high-fructose/high-glucose diet, suggesting that BLE as well as ELE inhibits the intestinal absorption of fructose. In fact, when rats were orally given BLE 10 min before the administration of fructose, the intestinal fructose absorption, as determined by measuring the elevated concentration of fructose in the portal vein, was significantly suppressed (unpublished data), in accordance with previous observations for ELE [15].

TBARS is a marker of lipid peroxidation. A “two-hit” hypothesis has been proposed for the pathogenesis of NASH [28], and oxidative stress is considered to be an important cause of the “second hit.” Markers of oxidative stress, including TBARS, are markedly elevated after fructose administration in rodents [11, 29]. In the present study, TBARS levels were significantly higher in the FG group than in the ST group, while they were significantly lower in the ELE and BLE groups than in the FG group. These results suggest that NASH induced by the high-fructose/high-glucose diet was associated with increased oxidative stress, an effect that was inhibited by ELE and BLE.

We observed by Western blot analysis that IL-6 expression levels were significantly higher in the FG group than in the ST group, while they were significantly lower in the ELE and BLE groups than in the FG group. IL-6 is hypothesized to sensitize the liver to injury, stimulate hepatocyte apoptosis, induce insulin resistance, and participate in NASH development [30]. Our results thus suggest that NASH brought on by a high-fructose/high-glucose diet is associated with increased IL-6 expression and that inhibition of NASH by ELE and BLE may be explained in part by decreased IL-6 expression.

It is well established that ELE and BLE contain a wide spectrum of polyphenols with strong antioxidative effects, such as hydrolysable tannins [17, 31]. Hydrolysable tannins and aglycones ameliorated lipopolysaccharide-induced liver injury by inhibition of inducible nitric oxide synthase (iNOS) expression in mice [32]. It has been reported that fructose-fed iNOS knockout mice did not exhibit increased levels of lipid peroxidation, phospho-I*κ*B and nuclear factor *κ*B activity, and TNF-*α* expression in the liver of rats [33]. We thus speculate that these physiological functions have a composite effect on the suppression of inflammation in the liver.

## 5. Conclusions

We show that ELE and BLE inhibit NASH induced by high-fructose/high-glucose diet in rats. This preventative effect is primarily associated with reduced lipogenesis, possibly due to the suppression of the intestinal fructose absorption. In addition, decreased oxidative stress and inflammatory cytokine expression might also provide a mechanism by which NASH is inhibited by ELE and BLE.

## Figures and Tables

**Table 1:** The composition of each experimental diet (g/1000 g diet).

	ST	FG	BLE	ELE
Cornstarch	700	0	0	0
Glucose	0	350	350	350
Fructose	0	350	350	350
Casein	170	170	170	170
Soybean oil	30	30	30	30
AIN-93G-MX mineral mix	35	35	35	35
Choline chloride	2	2	2	2
AIN-93VX vitamin mix	10	10	10	10
Methionine	3	3	3	3
Cellulose	50	50	40	40
BLE	0	0	10	0
ELE	0	0	0	10

BLE, banaba leaf extract; ELE, eucalyptus leaf extract.

**Table 2:** Food consumption, calorie intake, and body, liver, and EAT weight of rats.

	ST	FG	BLE	ELE
Food consumption (g)	750.2 ± 32.2	705.6 ± 41.4	678.3 ± 72.6^a^	678.0 ± 44.9^a^
Calorie intake (kcal)	2598.7 ± 111.4	2511.1 ± 147.3	2414.2 ± 258.3	2413.1 ± 159.6
Body weight (g)	358.2 ± 14.4	330.1 ± 10.5^a^	310.5 ± 29.8^a^	316.4 ± 24.4^a^
Liver weight (g)	10.05 ± 1.46	10.43 ± 0.38	8.97 ± 1.18^b^	9.92 ± 1.34
Liver/body weight ratio (%)	2.86 ± 0.23	3.16 ± 0.12^a^	2.88 ± 0.19^b^	3.13 ± 0.31
EAT weight (g)	4.95 ± 0.80	5.37 ± 1.11	4.18 ± 1.21^b^	4.13 ± 1.01^b^
EAT/body weight ratio (%)	1.41 ± 0.17	1.63 ± 0.35	1.33 ± 0.28	1.30 ± 0.27^b^

Data are presented as mean ± standard deviation.

^a^Significantly different from the ST group (*P* < 0.05). ^b^Significantly different from the FG group (*P* < 0.05).

EAT, epididymal adipose tissue.

**Table 3:** Serum data corresponding to each experimental group.

	ST	FG	BLE	ELE
AST (IU/L)	90.9 ± 14.6	91.1 ± 8.3	85.8 ± 7.8	87.1 ± 11.1
ALT (IU/L)	22.6 ± 6.7	28.7 ± 6.1^a^	20.0 ± 1.4^b^	23.7 ± 2.8
ALP (IU/L)	537.4 ± 153.6	552.4 ± 134.9	560.8 ± 128.5	518.6 ± 164.0
ChE (IU/L)	2.71 ± 0.95	2.89 ± 0.78	2.00 ± 0.89	2.43 ± 1.27
T-Cho (mg/dL)	92.8 ± 5.4	103.9 ± 15.7	84.1 ± 10.0^b^	97.1 ± 18.2
HDL-Cho (mg/dL)	28.0 ± 2.1	30.8 ± 3.0	28.0 ± 2.9	30.1 ± 3.8
AI	2.28 ± 0.08	2.37 ± 0.20	2.00 ± 0.20^b^	2.21 ± 0.33
Glucose (mg/dL)	114.3 ± 26.9	117.2 ± 22.3	117.0 ± 10.3	105.0 ± 4.8
Insulin (ng/mL)	1.56 ± 0.68	1.53 ± 0.99	1.21 ± 0.50	1.16 ± 0.14
Adiponectin (*μ*g/mL)	4.97 ± 1.30	4.78 ± 0.97	4.43 ± 0.98	4.79 ± 0.39

Data are presented as mean ± standard deviation.

^a^Significantly different from the ST group (*P* < 0.05). ^b^Significantly different from the FG group (*P* < 0.05).

AI, arteriosclerotic index; ALP, alkaline phosphatase; ALT, alanine aminotransferase; AST, aspartate aminotransferase; ChE, cholinesterase; HDL-Cho, high density lipoprotein; T-Cho, total cholesterol.

**Table 4:** Histological findings for each experimental group.

	ST	FG	BLE	ELE
Macrovesicular steatosis	0 (0-1)	2 (1–3)^a^	0 (0-1)^b^	0 (0-1)^b^
Microvesicular steatosis	2 (1–3)	2 (1–3)	1 (0-1)^a,b^	1 (1-2)^b^
Lobular inflammation	2 (0–2)	2 (1-2)	1 (0–2)	2 (1-2)
Portal inflammation	1 (1-1)	1 (1-1)	1 (0-1)	1 (1-1)
Lipogranulomas	0 (0-0)	2 (1–3)^a^	0 (0-1)^b^	0 (0-0)^b^
Portal fibrosis	0 (0-0)	1 (0-1)^a^	0 (0-1)	0 (0-1)
Perisinusoidal fibrosis	0 (0-0)	1 (1-1)^a^	0 (0-1)^b^	0 (0-1)^b^
Fibrosis stage	0 (0-0)	2 (1-2)^a^	0 (0–2)	1 (0–2)^a^

Data are presented as the median (min.–max.).

^a^Significantly different from the ST group (*P* < 0.05). ^b^Significantly different from the FG group (*P* < 0.05).
